# Integrating pretreatment CT radiomics and circulating tumor cells using machine learning to predict survival in hepatocellular carcinoma

**DOI:** 10.3389/fphar.2026.1788180

**Published:** 2026-05-19

**Authors:** Yongzhong Li, Shuixia Liu, Yanli Zeng, Yi Jiang, Zhaoyang Wang, Lianbin Wen, Yanqiong Song, Jianwen Zhang, Fei Wang

**Affiliations:** 1 Department of Oncology, Luxian People’s Hospital, Luzhou, China; 2 Department of Ophthalmology, Daping Hospital, Army Medical University, Chongqing, China; 3 Department of Oncology, Chongqing General Hospital, Chongqing University, Chongqing, China; 4 Department of Traditional Chinese Medicine (Oncology), RenShou County Hospital of Traditional Chinese Medicine, Meishan, Sichuan, China; 5 Institute of Medical Imaging and Artificial Intelligence, Jiangsu University, Zhenjiang, China; 6 Department of Geriatric Cardiology, Sichuan Academy of Medical Sciences & Sichuan Provincial People’s Hospital, Chengdu, China; 7 Department of Radiotherapy, Sichuan Cancer Hospital and Institute, Sichuan Cancer Center, School of Medicine, University of Electronic Science and Technology of China, Chengdu, China; 8 Department of Oncology, The Affiliated Hospital, Southwest Medical University, Luzhou, Sichuan, China; 9 Department of General Surgery, Luxian People’s Hospital, Luzhou, China

**Keywords:** circulating tumor cells, hepatocellular carcinoma, machine learning, radiomics, survival

## Abstract

**Background:**

Immunotherapy has shown promising potential in the treatment of advanced hepatocellular carcinoma (HCC). This study aimed to establish and validate a multimodal prognostic model based on clinical variables, CT radiomics, and circulating tumor cell (CTC) counts for survival prediction in advanced HCC.

**Methods:**

Pretreatment CT images and baseline clinical data were collected from patients with advanced HCC. Radiomic features were extracted from CT images, and candidate machine learning pipelines were screened to derive an optimal radiomics signature. Clinical and biomarker variables associated with overall survival were identified using Cox regression analyses and incorporated into prognostic nomograms. Model discrimination, calibration, and clinical utility were evaluated in internal and external validation cohorts.

**Results:**

The addition of immunotherapy was associated with improved prognosis in patients with advanced HCC. Among the prognostic models, the Clinical–Radiomic–CTC nomogram outperformed the Clinical–Radiomic nomogram, showing a higher concordance index (0.789) and higher area under the receiver operating characteristic curves (AUCs) for 1-, 2-, and 3-year OS (0.889, 0.771, and 0.838, respectively).

**Conclusion:**

Machine learning models integrating radiomics and CTCs provided robust individualized prognostic prediction, supporting risk stratification and clinical decision-making.

## Introduction

Hepatocellular carcinoma (HCC) remains one of the most prevalent and lethal malignancies worldwide. Although advances in surveillance, early detection, and treatment have improved outcomes for selected patients, the majority are still diagnosed at an advanced stage, leading to limited therapeutic options and poor survival (Yang et al., 2024). Therefore, more effective treatment strategies are urgently needed for advanced HCC.

Radiotherapy combined with tyrosine kinase inhibitors (TKIs) (R + T) has shown encouraging efficacy in advanced HCC. A meta-analysis including 46 studies (7595 patients) reported that sorafenib plus radiotherapy was associated with the most favorable overall survival (OS) and progression-free survival (PFS) compared with other monotherapies or combination regimens (Li et al., 2023). In addition, a phase III randomized controlled trial demonstrated that stereotactic body radiotherapy (SBRT) plus sorafenib significantly prolonged median PFS (mPFS) compared with sorafenib alone (9.2 vs. 5.5 months, P < 0.001). However, the improvement in median OS (mOS) did not reach statistical significance (15.8 vs. 12.3 months, P = 0.06). Notably, multivariable Cox regression indicated that SBRT was independently associated with improved OS (hazard ratio [HR] 0.72; 95% confidence interval [CI] 0.52–0.99; P = 0.04) (Dawson et al., 2025). Despite these benefits, further survival gains remain necessary.

Immune checkpoint inhibitors, particularly programmed cell death protein 1 (PD-1) inhibitors (P), have expanded therapeutic options for advanced HCC (Llovet et al., 2024). The IMbrave150 study showed that atezolizumab plus bevacizumab extended mOS to 19.2 months in unresectable HCC, with an objective response rate (ORR) of 27.3% (Finn et al., 2020; Cheng et al., 2022). These findings support the rationale for integrating immunotherapy into multimodal treatment strategies.

Given the heterogeneity of treatment response and disease trajectories, effective and non-invasive monitoring tools are essential to assess therapeutic efficacy and disease progression (Barcena-Varela et al., 2025). Non-invasive approaches have gained increasing attention because they enable longitudinal monitoring without repeated biopsies (Li et al., 2024). Radiomics—high-throughput extraction of quantitative features from medical images—can capture tumor phenotype, microenvironmental information, and intratumoral heterogeneity (Sagir, 2020). In parallel, liquid biopsy approaches such as circulating tumor cell (CTC) detection can provide real-time insights into tumor burden, metastatic potential, and treatment response (Zhao et al., 2023; Ahn et al., 2021). Integrating radiomics with CTC data may therefore offer complementary, multi-dimensional evaluation of tumor biology and clinical outcomes (Jiao et al., 2022).

In this study, we investigated whether adding PD-1 inhibitors to R + T (R + T + P) could further improve survival in patients with advanced HCC. In addition, we developed predictive models for patients receiving R + T + P by integrating radiomics, CTC counts, and baseline clinical variables using machine learning algorithms.

## Materials and methods

### Eligibility and study population

This multicenter study enrolled 295 patients with advanced HCC from five centers, including 172 treated with radiotherapy plus TKIs plus PD-1 inhibitors (R + T + P) and 123 treated with radiotherapy plus TKIs (R + T). This study was approved by the Luxian People’s Hospital. Written informed consent was obtained from all patients.

Inclusion criteria were: (1) age ≥18 years; (2) Eastern Cooperative Oncology Group performance status (ECOG PS) of 0–1; and (3) Barcelona Clinic Liver Cancer (BCLC) stage B or C. Exclusion criteria included: (1) diffuse HCC; (2) Child–Pugh class C; and (3) hepatic encephalopathy or refractory ascites.

### Treatment

All patients received intensity-modulated radiotherapy (IMRT) in combination with TKIs. The addition of PD-1 inhibitors was recommended by treating clinicians, and the final decision was made jointly with the patient. PD-1 inhibitors and TKIs were continued until unacceptable toxicity or progressive disease (PD) occurred. Drug dosing was individualized according to clinical practice.

### Follow-up

PFS was defined as the time from treatment initiation to PD or death. OS was defined as the time from treatment initiation to death or last follow-up.

### CTC, radiomics features, and machine learning

Among patients receiving R + T + P, 112 had available pretreatment CTC counts. Pretreatment peripheral blood samples for CTC detection were collected within 1 week before treatment initiation. For model development in the R + T + P cohort with CTC data, the 112 patients were randomly split into a training set (n = 76) and an internal validation set (n = 36) at a 7:3 ratio. In addition, pretreatment imaging data from 41 independent patients treated with R + T + P at another institution were collected as an external validation cohort.

Gross tumor volume (GTV) segmentation was manually performed on pretreatment CT images by one oncologist and one radiologist, who jointly delineated the tumor region. Both readers were blinded to patient outcomes during the segmentation process. Radiomic features were then extracted from the manually delineated GTV on pretreatment CT images using 3D Slicer. The extracted feature families included first-order, shape, and texture features, including GLCM, GLDM, GLRLM, GLSZM, and NGTDM features. A total of 1130 radiomic features were extracted. No z-score standardization was performed during feature extraction or model development. Subsequently, 101 candidate machine-learning–based radiomics pipelines were evaluated, and the optimal model was selected to generate an individualized radiomics score (Rad-score).

### AI-model development and evaluation

Univariate and multivariate Cox regression analyses were performed in the training set to identify clinical prognostic factors associated with OS. Two nomograms were constructed: (Yang et al., 2024): a Clinical–Radiomic nomogram incorporating baseline variables plus radiomics; and (Li et al., 2023) a Clinical–Radiomic–CTC nomogram integrating baseline variables, radiomics, and CTC counts.

Model performance was assessed using receiver operating characteristic–area under the curve (ROC-AUC), decision curve analysis (DCA), calibration plots, partial dependence plots (PDP), and SHapley Additive exPlanations (SHAP) in internal and external validation cohorts, as applicable.

### Statistical analysis

Categorical variables were compared using the chi-square test or Fisher’s exact test, whereas continuous variables were compared using the Student’s t-test or Mann–Whitney U test. OS and PFS were estimated using the Kaplan-Meier method and compared using log-rank tests. All analyses were performed using R software (version 4.4.3). A two-sided P < 0.05 was considered statistically significant.

## Results

### Patient characteristics

A total of 295 patients with advanced HCC were included (R + T + P, n = 172; R + T, n = 123). Baseline characteristics were comparable between the two groups (Table 1).

**TABLE 1:** Comparison of R + T vs. R + T + P in 295 advanced HCC patients.

Variables	R + T	R + T + P	P
	No. (%)	No. (%)	
Total	123	172	​
Male	99 (80.5)	148 (86.0)	0.265
Age (mean ± SD, years)	54.0 ± 10.5	54.3 ± 10.2	0.857
Tumor number ≥2	87 (70.7)	111 (64.5)	0.321
Size (mean ± SD, cm)	7.17 (3.79)	7.57 (4.27)	0.391
HBV	89 (72.4)	108 (62.8)	0.097
Child	​	​	0.174
A	100 (81.3)	127 (73.8)	​
B	23 (18.7)	45 (26.2)	​
BCLC	​	​	0.392
B	22 (17.9)	39 (22.7)	​
C	101 (82.1)	133 (77.3)	​
PVTT	87 (70.7)	110 (64.0)	0.273
Lymph node metastasis	48 (39.0)	82 (47.7)	0.175
Extrahepatic metastases	37 (30.1)	38 (22.1)	0.156
Lung	17 (13.8)	17 (9.88)	0.390
Bone	16 (13.0)	22 (12.8)	1.000
Other	12 (9.76)	9 (5.23)	0.208
Leukocyte (mean ± SD, 1000/µL)	5.47 ± 2.09	6.26 ± 2.76	0.108
<4000/µL	31 (25.2)	29 (16.9)	​
≥4000/µL	92 (74.8)	143 (83.1)	​
PLT ≥ (mean ± SD, 1000/µL)	146 ± 68.0	163 ± 76.0	0.158
<100000/µL	33 (26.8)	33 (19.2)	​
≥100000/µL	90 (73.2)	139 (80.8)	​
ALT (mean ± SD, U/L)	51.7 ± 84.5	62.1 ± 70.6	0.080
<40 U/L	75 (61.0)	86 (50.0)	​
≥40 U/L	48 (39.0)	86 (50.0)	​
AFP (mean ± SD, ng/mL)	698 ± 758	512 ± 693	0.185
<200	54 (43.9)	98 (57.0)	​
≥200, <400	8 (6.50)	9 (5.23)	​
≥400	61 (49.6)	65 (37.8)	​

R, radiotherapy; T, tyrosine kinase inhibitors; P, PD-1, inhibitors; HCC, hepatocellular carcinoma; HBV, hepatitis B virus; BCLC, barcelona clinic liver cancer; PVTT, portal vein tumor thrombosis; PLT, platelet; ALT, alanine aminotransferase; AFP, alpha-fetoprotein.

### Survival outcomes

**FIGURE 1:**
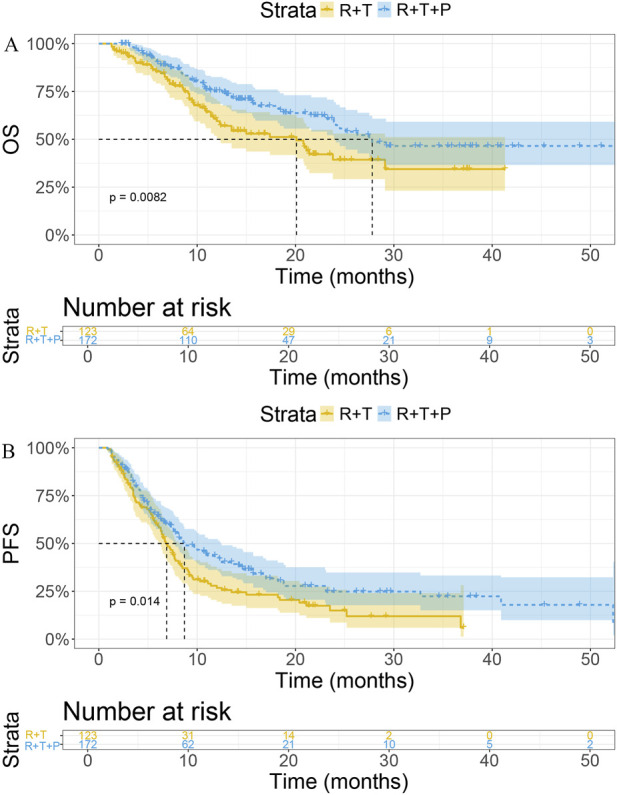
The R + T + P group exhibited longer mOS **(A)** and mPFS **(B)** compared to the R + T group. Abbreviations: R, radiotherapy; T, tyrosine kinase inhibitors; P, PD-1 inhibitors; mOS, median overall survival; mPFS, median progression-free survival.

Compared with the R + T group, the R + T + P group achieved significantly longer mOS (27.8 vs. 20.1 months, P = 0.008; Figure 1A) and mPFS (8.7 vs. 6.9 months, P = 0.014; Figure 1B).

### Cox regression analyses for OS

Univariate and multivariate Cox analyses indicated that receiving R + T + P, having fewer than two tumors, smaller tumor size, and absence of portal vein tumor thrombosis (PVTT) were associated with improved OS (Supplementary Table S1).

### Radiomics modeling and rad-score development

**FIGURE 2:**
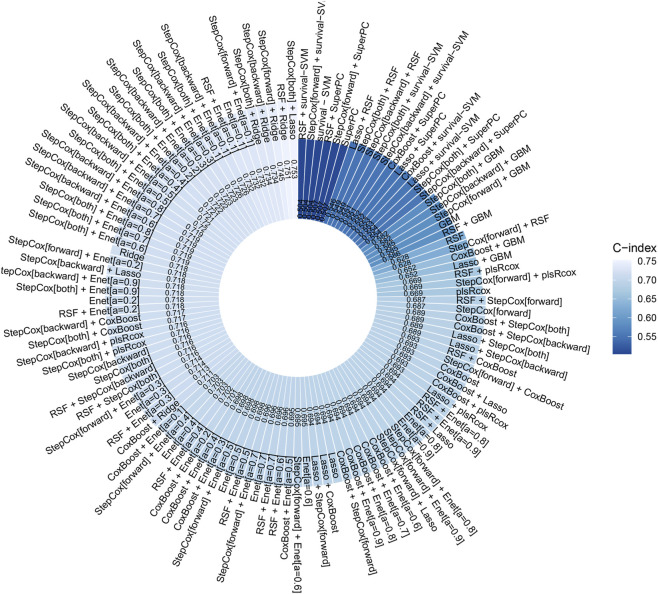
The C-index for the 101 machine learning algorithms was calculated to assess their performance in predicting overall survival based on radiomics features.

Using 1130 radiomic features and 101 candidate machine-learning pipelines, the StepCox + Lasso approach achieved the highest concordance index (C-index = 0.753; Figure 2). A radiomics-derived risk score was calculated for each patient. Using the median risk score (0.757) as the cutoff, patients were stratified into high- and low-risk groups. The high-risk group had significantly worse OS than the low-risk group (18.2 months vs. not reached, P < 0.001; Supplementary Figure S1A). ROC-AUC values for predicting 1-, 2-, and 3-year OS were 0.755, 0.739, and 0.765, respectively (Supplementary Figure S1B).

### Nomogram construction and validation

OS and PFS were not significantly different among these cohorts (OS: P = 0.59, Supplementary Figure S2A; PFS: P = 0.60; Supplementary Figure S2B). In the training cohort, multivariate Cox analysis identified PVTT, aspartate aminotransferase (AST), and alpha-fetoprotein (AFP) as independent predictors of OS in patients receiving R + T + P (Supplementary Table S2).

**FIGURE 3:**
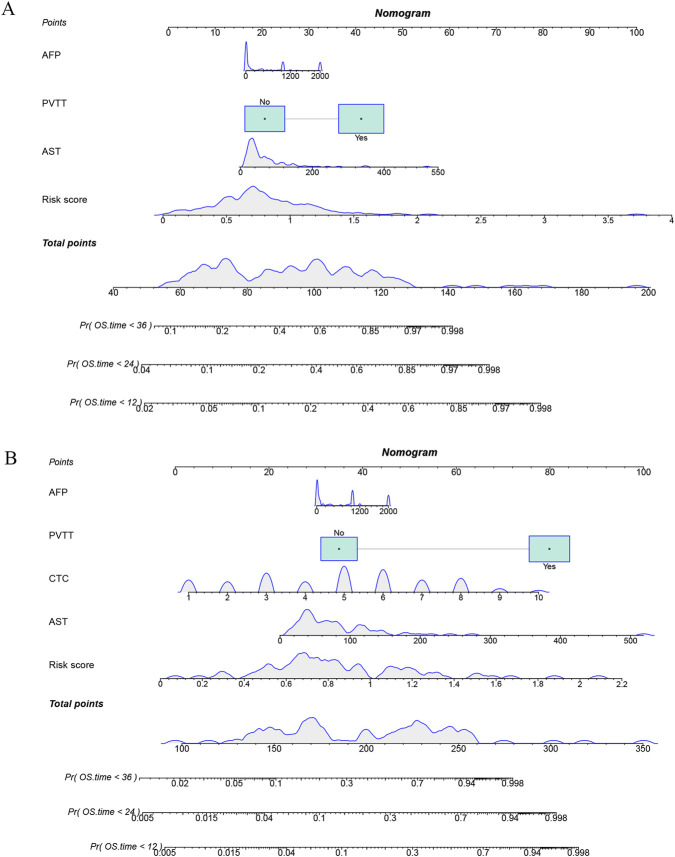
Based on these three clinical risk factors, radiomic risk scores, and CTC count, two nomograms were constructed: Clinical-Radiomic Nomogram **(A)**, and Clinical-Radiomic-CTC Nomogram **(B)**. Abbreviations: CTC, circulating tumor cells.

Three nomograms were then developed: Clinical–Radiomic nomogram (Figure 3A), and Clinical–Radiomic–CTC nomogram (Figure 3B). The corresponding C-indices were 0.773, and 0.789, respectively.

**FIGURE 4:**
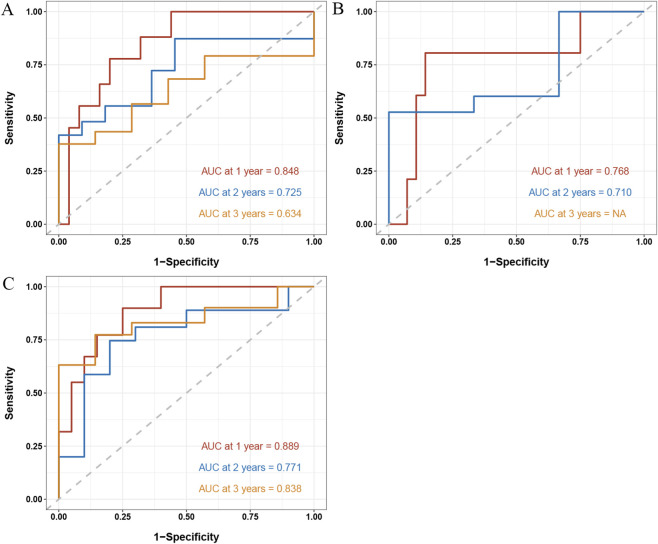
The ROC-AUC values for predicting 1-, 2-, and 3-year overall survival were as follows: , Clinical-Radiomic Nomogram (A, internal; B, external), and Clinical-Radiomic-CTC Nomogram (C, internal). Abbreviations: ROC-AUC, receiver operating characteristic-area under the curve; CTC, circulating tumor cells.

For the Clinical–Radiomic nomogram, ROC-AUC values for 1-, 2-, and 3-year OS prediction were 0.848, 0.725, and 0.634 in the internal validation cohort (Figure 4A), and 0.768, 0.710, and not available (NA) in the external validation cohort (Figure 4B). For the Clinical–Radiomic–CTC nomogram, internal validation ROC-AUC values were 0.889, 0.771, and 0.838 (Figure 4C).

**FIGURE 5:**
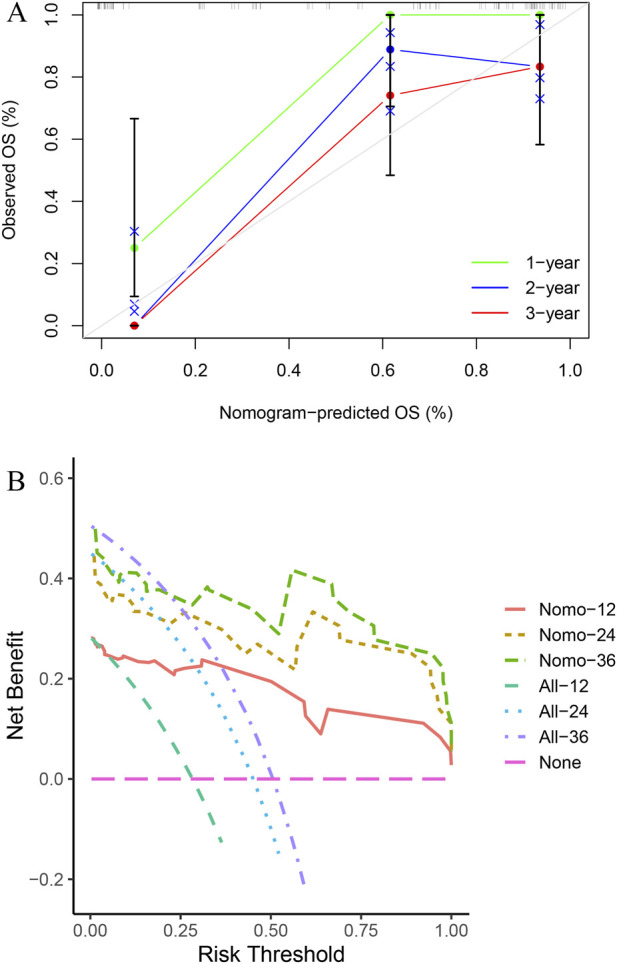
The internal validation set confirmed the good predictive performance of the Clinical-Radiomic-CTC Nomogram, as shown by the calibration **(A)** and DCA curves **(B)**. Abbreviations: CTC, circulating tumor cells, DCA, Decision Curve Analysis.

Calibration and DCA curves supported good predictive performance of the Clinical–Radiomic nomogram in both internal and external validation cohorts (Supplementary Figures S3A,B; Supplementary Figures S4A,B). The Clinical–Radiomic–CTC nomogram also showed good calibration and net benefit in the internal validation cohort (Figures 5A,B).

Model interpretability analyses demonstrated that PVTT was the most influential predictor of OS, followed by CTC count, radiomics risk score, AST, and AFP (Supplementary Figure S5). PDP further confirmed that absence of PVTT, lower AST, lower AFP, lower CTC, and lower radiomics risk score were associated with improved OS (Supplementary Figure S6).

## Discussion

To our knowledge, this is also the first study to evaluate R + T + P using an integrated framework combining machine learning, radiomics, CTC counts, and baseline clinical data. The observed survival benefit may be explained by immune-related mechanisms underlying synergistic interactions among radiotherapy, targeted therapy, and immune checkpoint blockade (Cho et al., 2019; Xu et al., 2025). PD-1 inhibitors can reinvigorate exhausted T cells and enhance anti-tumor immune surveillance, potentially amplifying radiation-induced antigen release and improving tumor control when combined with TKIs (Zhang et al., 2023; Yamaguchi et al., 2022; Chen et al., 2018). Landmark trials have highlighted the promise of combination strategies in HCC (Hall et al., 2024; Zhong et al., 2021). IMbrave150 demonstrated survival benefit with atezolizumab plus bevacizumab (Finn et al., 2020). Meanwhile, SBRT plus sorafenib improved mPFS but did not significantly improve mOS in a phase III trial (Dawson et al., 2025). Other trials have also suggested that multi-agent approaches may be required to achieve meaningful outcome improvements in unresectable disease (Kudo et al., 2025). Collectively, our findings support R + T + P as a novel and promising regimen for advanced HCC.

Importantly, our predictive modeling results indicate that integrating multi-modal data improves prognostic performance. The Clinical–Radiomic–CTC nomogram outperformed the Clinical–Radiomic model, as reflected by its higher C-index and ROC-AUC values. This highlights the value of combining radiomics and liquid biopsy biomarkers with clinical variables to enable more accurate individualized risk stratification and treatment guidance (Xia et al., 2023; Sun et al., 2021; Ndow et al., 2023; Wang et al., 2022).

Beyond performance, interpretability is crucial for clinical translation. Using SHAP and PDP, we identified PVTT, CTC counts, radiomics risk scores, AST, and AFP as key contributors to outcome prediction (Su et al., 2022; Shen et al., 2021; Zhou et al., 2023; Wang et al., 2023; Huang et al., 2016). Radiomics offers a non-invasive approach to quantify tumor heterogeneity—often linked to treatment resistance and adverse prognosis—while CTC enumeration provides real-time biological information reflecting tumor burden and metastatic potential (Namba et al., 2025). Integrating these complementary signals into a unified, explainable framework may help bridge the gap between predictive modeling and clinical decision-making. From a clinical perspective, the Clinical–Radiomic–CTC nomogram may serve as a practical pretreatment risk-stratification tool for patients with advanced HCC considered for immunoradiotherapy-based combination treatment. Because the model integrates routinely available clinical variables with noninvasive imaging features and liquid biopsy data, it may help identify patients at higher risk of poor survival before treatment initiation. Such patients may benefit from closer monitoring, intensified follow-up, individualized therapeutic adjustment, or consideration for enrollment in prospective clinical trials. In contrast, patients classified as low risk may be more likely to achieve favorable outcomes under the current treatment strategy. Therefore, this model has potential value in supporting risk-adapted management and individualized prognostic assessment in real-world practice.

From a methodological perspective, machine learning provides a data-driven framework to integrate heterogeneous, high-dimensional inputs—particularly radiomic features and CTC counts—into a unified prognostic signature, which is difficult to achieve with conventional regression alone (Shan et al., 2025; Huang et al., 2024). Radiomics typically yields hundreds to thousands of correlated features, and direct inclusion of these variables in traditional models may lead to multicollinearity and overfitting, especially in moderate sample sizes (Jin et al., 2024). By leveraging regularization-based survival learning and systematic model screening, we were able to identify a compact, reproducible radiomics-derived risk score and subsequently incorporate it with clinically interpretable variables (PVTT, AST, AFP) and CTC counts to build an individualized prediction tool. Importantly, the incremental gain observed in the Clinical–Radiomic–CTC nomogram supports the complementary value of imaging phenotypes and circulating biomarkers, suggesting that multi-modal ML models can more comprehensively capture tumor aggressiveness, microenvironmental context, and metastatic potential (He et al., 2025). To facilitate clinical translation, we further adopted explainable AI strategies (SHAP and PDP) to quantify feature contributions and visualize risk relationships, thereby improving transparency and enabling clinicians to verify whether model behavior aligns with biological and clinical plausibility. Collectively, these findings indicate that ML-enabled, noninvasive multi-modal modeling may serve as a practical approach for pre-treatment risk stratification, individualized prognosis estimation, and decision support in patients receiving immunoradiotherapy-based combination regimens (Feng et al., 2025).

This study has several limitations. First, because of its retrospective and non-randomized design, treatment allocation was influenced by clinician recommendation and patient preference, introducing potential selection bias and confounding by indication. Thus, the survival differences observed between groups should be interpreted cautiously. Second, PD-1 inhibitor and TKI regimens were not fully standardized across centers, which may have introduced treatment heterogeneity. Third, inter-institutional differences in patient characteristics and clinical practice may have limited the generalizability of the results. Fourth, inter-institutional differences in patient characteristics and clinical practice may have further limited the generalizability of the results. Future prospective studies with larger cohorts and more standardized treatment, imaging, and assessment protocols are needed to validate these findings.

Radiotherapy combined with PD-1 inhibitors and TKIs significantly improves survival outcomes in patients with advanced HCC. Furthermore, integrating clinical variables, radiomics, and CTC counts through machine learning provides an effective strategy for individualized prognostic assessment and risk stratification. These results support R + T + P as a promising therapeutic option and underscore the value of AI-assisted decision-making in optimizing treatment strategies for advanced HCC.

## Data Availability

The original contributions presented in the study are included in the article/Supplementary Material, further inquiries can be directed to the corresponding author.
