# Supplementary material for: Integrating pretreatment CT radiomics and circulating tumor cells using machine learning to predict survival in hepatocellular carcinoma
Source: Front Pharmacol. 2026 May 19;17:1788180. doi: 10.3389/fphar.2026.1788180 (PMC13227010; doi:10.3389/fphar.2026.1788180)

| **Supplementary Table 1**. Univariate and multivariate Cox regression analysis of overall survival | | | |
| --- | --- | --- | --- |
|  | | HR (univariable) | HR (multivariable) |
| Treatment | R + T |  |  |
|  | R + T + P | 0.61 (0.42-0.88, p=.009) | 0.67 (0.45-0.99, p=.043) |
| Age |  | 1.00 (0.98-1.02, p=.901) |  |
| Tumor number | ＜ 2 |  |  |
|  | ≥ 2 | 1.83 (1.19-2.81, p=.006) | 1.62 (1.04-2.54, p=.035) |
| Size |  | 1.10 (1.05-1.14, p<.001) | 1.05 (1.00-1.10, p=.048) |
| Sex | Female |  |  |
|  | Male | 0.94 (0.56-1.55, p=.801) |  |
| HBV | No |  |  |
|  | Yes | 1.54 (1.02-2.31, p=.038) | 1.36 (0.90-2.07, p=.145) |
| Child | A |  |  |
|  | B | 1.62 (1.05-2.49, p=.029) | 1.39 (0.88-2.21, p=.157) |
| BCLC | B |  |  |
|  | C | 3.03 (1.58-5.80, p<.001) | 1.43 (0.65-3.14, p=.375) |
| PVTT | No |  |  |
|  | Yes | 3.42 (2.12-5.52, p<.001) | 1.97 (1.10-3.56, p=.024) |
| Lymph node metastasis | No |  |  |
|  | Yes | 1.19 (0.82-1.72, p=.365) |  |
| Extrahepatic metastases | No |  |  |
|  | Yes | 1.63 (1.11-2.40, p=.012) | 1.54 (1.00-2.38, p=.051) |
| Leukocyte |  | 1.00 (0.93-1.08, p=.927) |  |
| PLT |  | 1.00 (1.00-1.00, p=.593) |  |
| ALT |  | 1.00 (1.00-1.00, p=.957) |  |
| AFP |  | 1.00 (1.00-1.00, p<.001) | 1.00 (1.00-1.00, p=.093) |
| R, radiotherapy; T, tyrosine kinase inhibitors; P, PD-1 inhibitors; HBV, hepatitis B virus; BCLC, barcelona clinic liver cancer; PVTT, portal vein tumor thrombosis; PLT, platelet; ALT, alanine aminotransferase; AFP, alpha-fetoprotein. | | | |

| **Supplementary Table 2**. Univariate and multivariate Cox regression analysis of overall survival in training set | | | |
| --- | --- | --- | --- |
|  | | HR (univariable) | HR (multivariable) |
| Age |  | 1.00 (0.98-1.03, p=.771) |  |
| Tumor number | ＜ 2 | Reference |  |
|  | ≥ 2 | 1.58 (0.82-3.05, p=.171) |  |
| Size |  | 1.10 (1.04-1.17, p=.002) | 1.02 (0.95-1.09, p=.649) |
| Sex | Female |  |  |
|  | Male | 0.88 (0.31-2.48, p=.804) |  |
| HBV | No |  |  |
|  | Yes | 1.56 (0.86-2.81, p=.142) |  |
| Child | A |  |  |
|  | B | 1.53 (0.79-2.96, p=.206) |  |
| BCLC | B |  |  |
|  | C | 2.96 (1.17-7.50, p=.022) | 1.53 (0.57-4.06, p=.398) |
| PVTT | No |  |  |
|  | Yes | 4.72 (2.27-9.81, p<.001) | 3.14 (1.38-7.11, p=.006) |
| Lymph node metastasis | No |  |  |
|  | Yes | 1.27 (0.71-2.26, p=.416) |  |
| Extrahepatic metastases | No |  |  |
|  | Yes | 1.28 (0.66-2.47, p=.464) |  |
| Leukocyte |  | 1.03 (0.93-1.14, p=.630) |  |
| Neutrophils |  | 1.04 (0.94-1.16, p=.440) |  |
| Hemoglobin |  | 0.99 (0.98-1.00, p=.068) |  |
| PLT |  | 1.00 (1.00-1.01, p=.169) |  |
| Total bilirubin |  | 1.00 (0.99-1.01, p=.702) |  |
| ALP |  | 1.00 (1.00-1.00, p=.005) | 1.00 (1.00-1.00, p=.494) |
| ALT |  | 1.00 (1.00-1.00, p=.977) |  |
| AST |  | 1.01 (1.00-1.01, p<.001) | 1.00 (1.00-1.01, p=.036) |
| Albumin |  | 0.95 (0.91-1.00, p=.037) | 0.99 (0.95-1.04, p=.755) |
| AFP |  | 1.00 (1.00-1.00, p<.001) | 1.00 (1.00-1.00, p=.007) |
| R, radiotherapy; T, tyrosine kinase inhibitors; P, PD-1 inhibitors; HBV, hepatitis B virus; BCLC, barcelona clinic liver cancer; PVTT, portal vein tumor thrombosis; PLT, platelet; ALP, alkaline phosphatase; ALT, alanine aminotransferase; AST, aspartate aminotransferase; AFP, alpha-fetoprotein. | | | |

**Supplementary Fig. 1** Based on a median risk score of 0.757, patients were divided into high and low expression groups, with the high expression group showing worse OS (A). The ROC curve for predicting OS based on the risk score (B).

Abbreviations: OS, median overall survival; ROC, receiver operating characteristic; AUC, area under the curve.


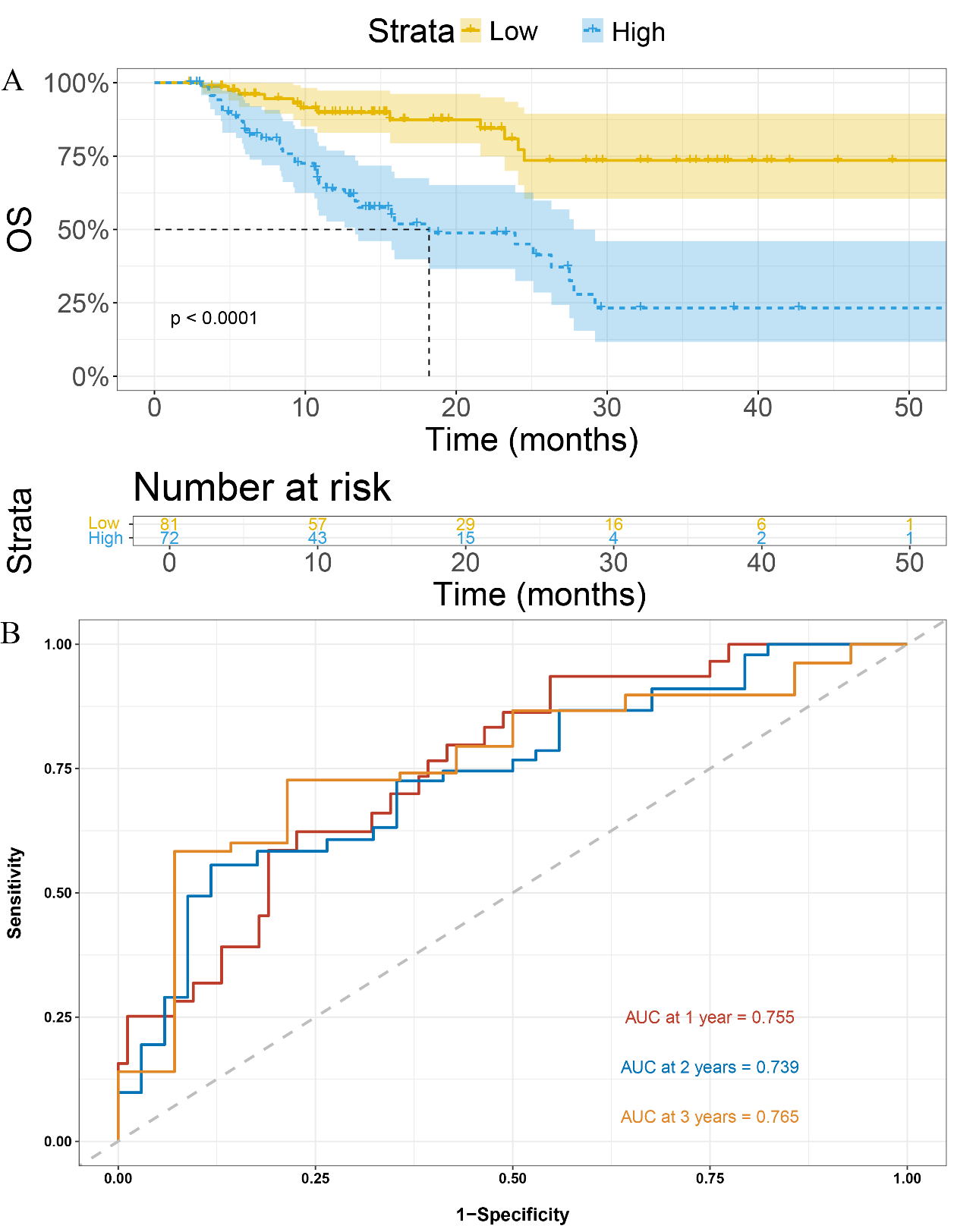


**Supplementary Fig. 2** No significant differences were observed in the mOS (A) and mPFS (B) among the training set, internal validation set, and external validation set.

**Abbreviations**: mOS, median overall survival; mPFS, median progression-free survival.


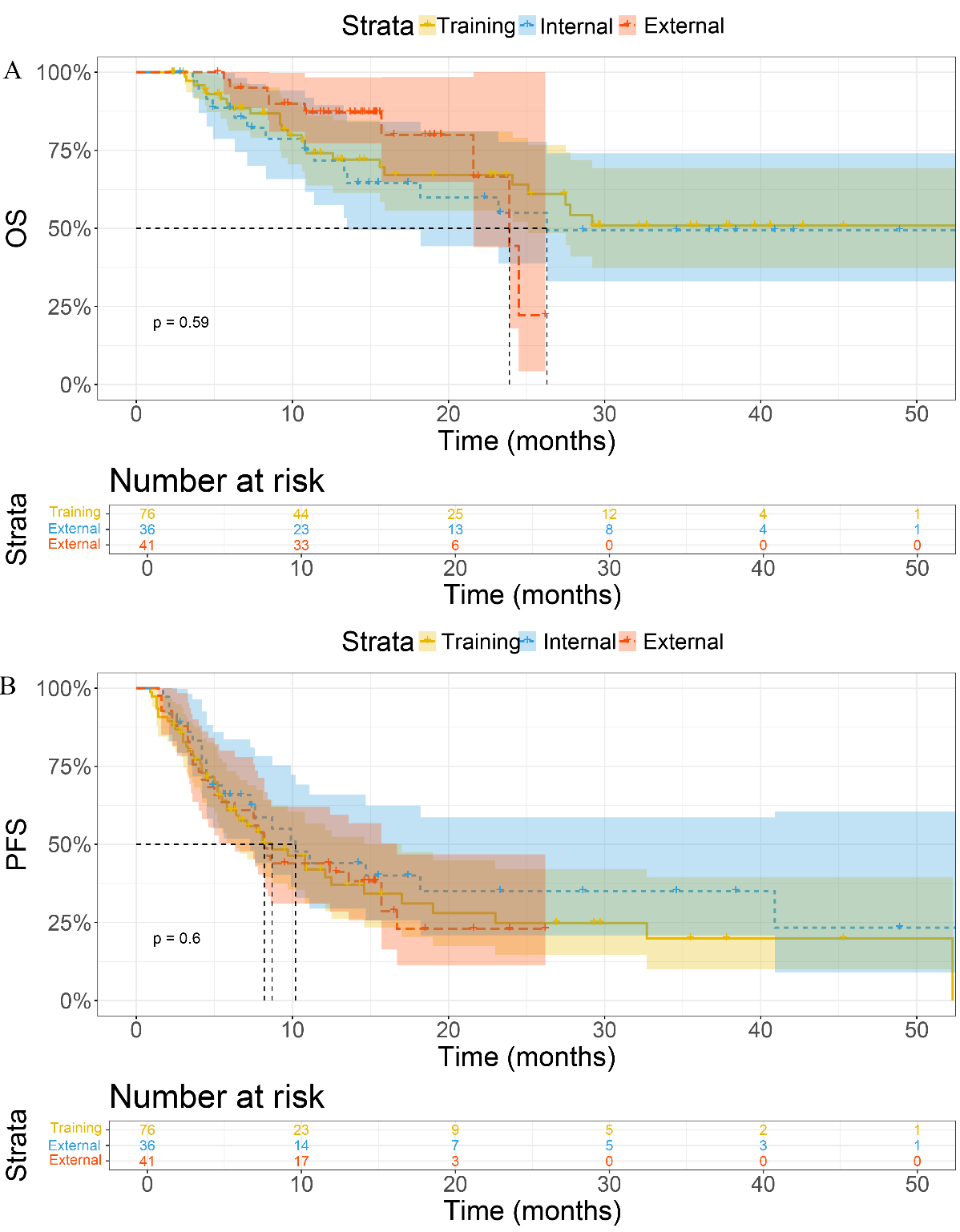


**Supplementary Fig. 3** In both the internal (A) and external validation sets (B), the calibration curve confirmed that the Clinical-Radiomic Nomogram exhibited good predictive performance.


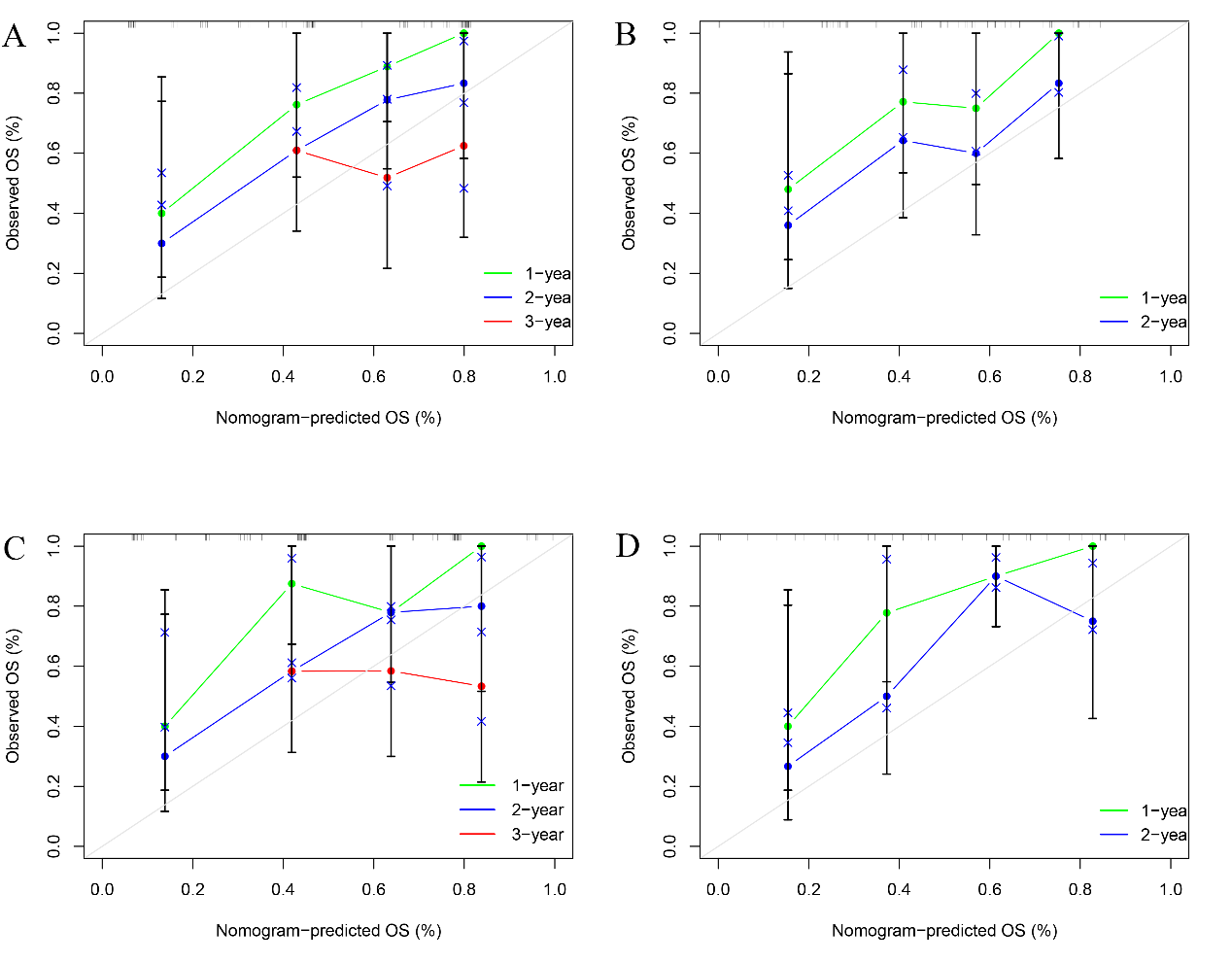


**Supplementary Fig. 4** In both the internal (A) and external validation (B) sets, the DCA curve confirmed that the Clinical-Radiomic Nomogram exhibited good predictive performance.

Abbreviations: DCA, Decision Curve Analysis.


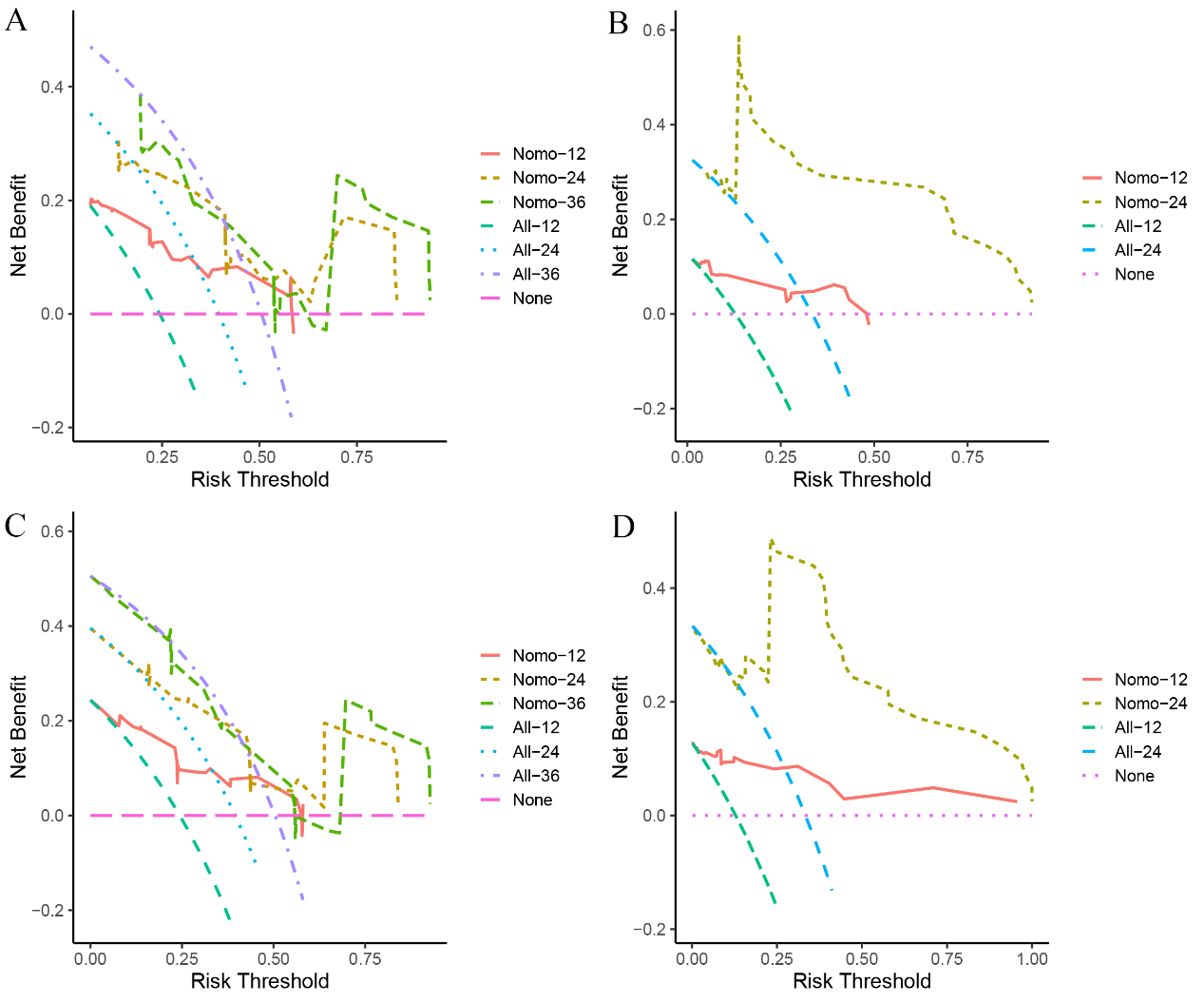


**Supplementary Fig. 5** In the training set, the SHAP identified PVTT as the most critical factor affecting overall survival in advanced HCC patients receiving R+T+P, followed by CTC, risk score, AST, and AFP.

Abbreviations: HCC, hepatocellular carcinoma; R, radiotherapy; T, tyrosine kinase inhibitors; P, PD-1 inhibitors; PVTT, portal vein tumor thrombosis; CTC, circulating tumor cell; AST, aspartate aminotransferase; AFP, alpha-fetoprotein.


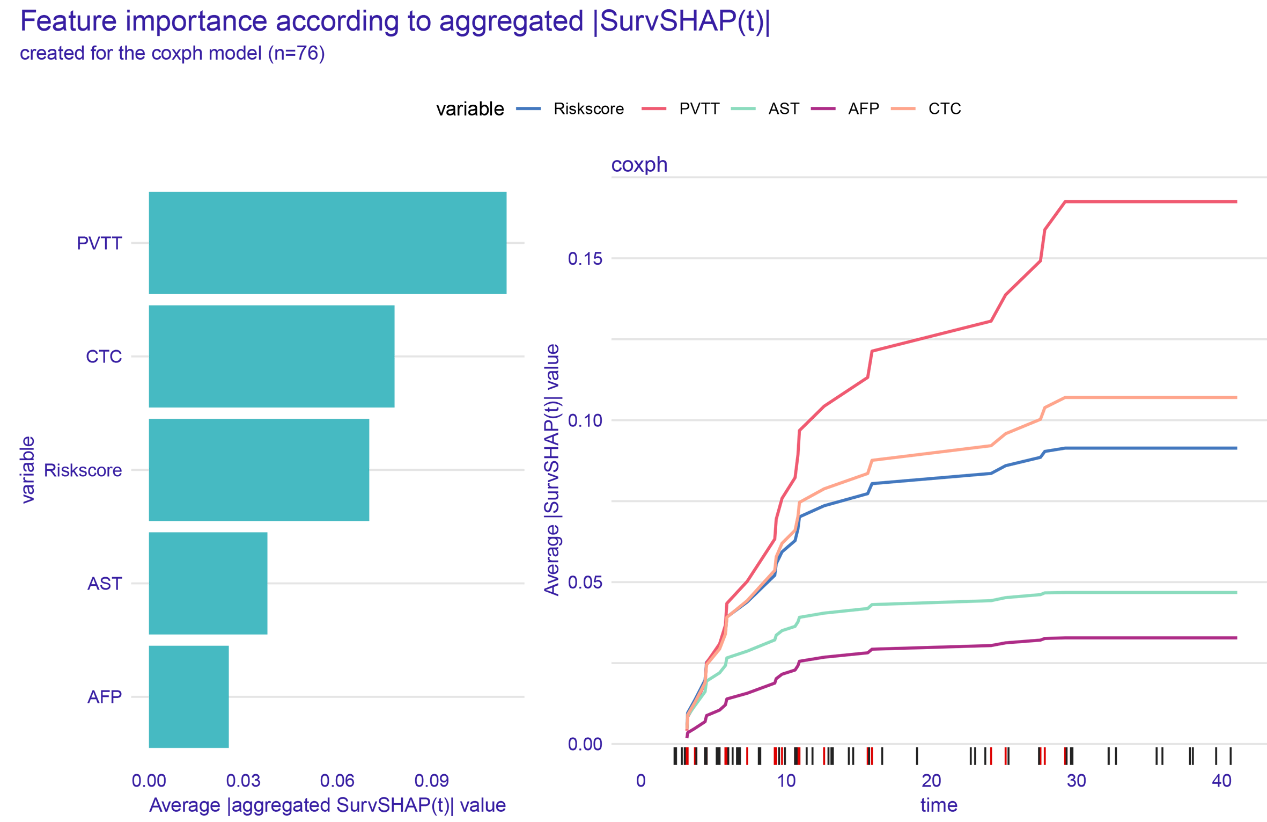


**Supplementary Fig. 6** The Partial Dependence Plots confirmed that the absence of PVTT, lower AST, lower AFP, lower CTC, and lower risk score were associated with improved overall survival.

Abbreviations: PVTT, portal vein tumor thrombosis; CTC, circulating tumor cell; AST, aspartate aminotransferase; AFP, alpha-fetoprotein.


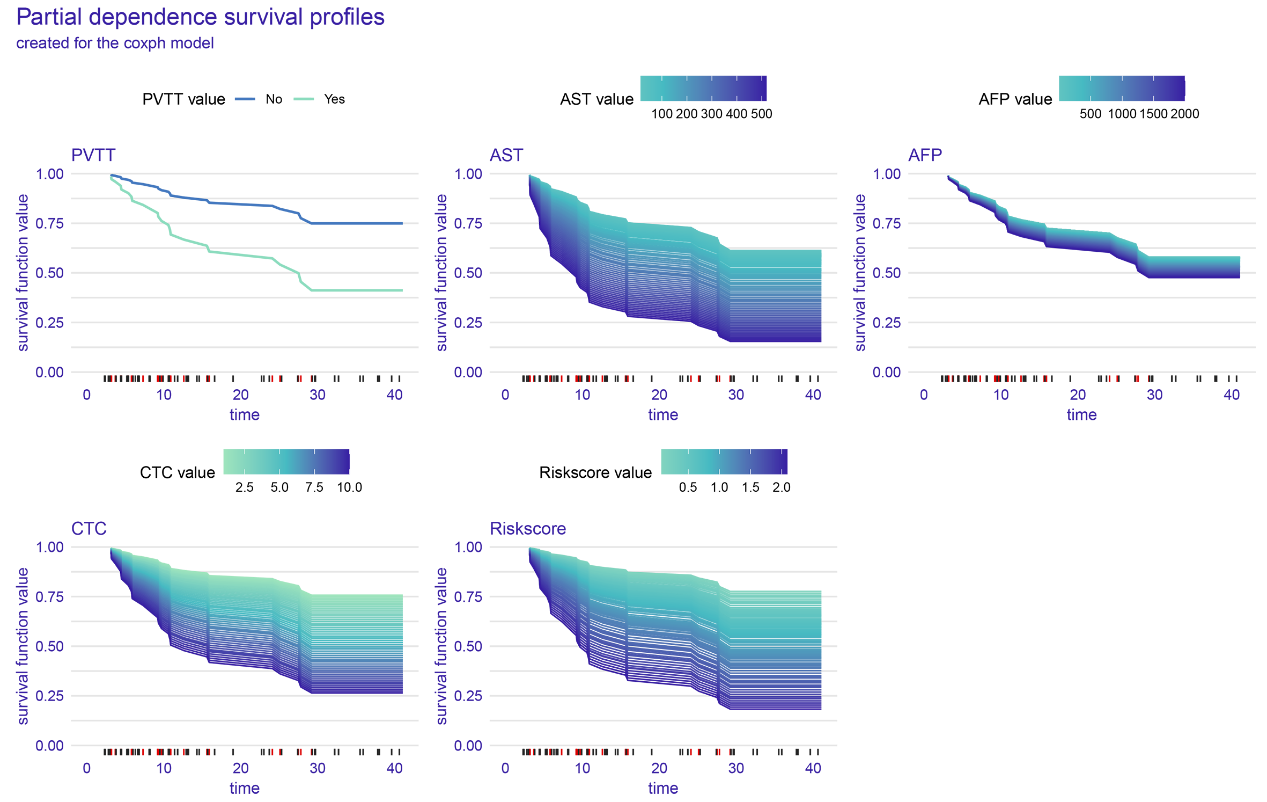

Supplement: Supplementary file 1 [file Supplementaryfile1.docx]
